# Group leaders establish cooperative norms that persist in subsequent interactions

**DOI:** 10.1371/journal.pone.0222724

**Published:** 2019-09-19

**Authors:** Ashley Harrell

**Affiliations:** Department of Sociology, Duke University, Durham, NC, United States of America; Massachusetts Institute of Technology, UNITED STATES

## Abstract

The temptation to free-ride on others’ contributions to public goods makes enhancing cooperation a critical challenge. Solutions to the cooperation problem have centered on installing a sanctioning institution where all can punish all, i.e., peer punishment. But a new, growing literature considers whether and when the designation of a group leader—one group member, given the sole ability to administer punishment—is an effective and efficient alternative. What remains unknown is whether and to what extent these group leaders establish cooperative norms in their groups via their own contributions to the public good, their use of sanctions, or both. Nor has past work examined whether leaders’ behaviors have lasting effects on non-leaders’ cooperation in subsequent interactions, outside of the leader’s purview. Here I show that leaders’ contributions to the public good predict non-leaders’ subsequent cooperation. Importantly, the effect is not limited to cooperation within the institution: the effect of leaders’ contributions continue to predict non-leaders’ contributions in a later interaction, where sanctions are removed. This process is mediated by non-leaders’ increased contributions in the institution, suggesting that leaders have effects on followers that shape followers’ subsequent behaviors. These effects occur above and beyond a baseline tendency to be influenced by non-leader group members; they also occur above and beyond the influence of peers in groups under a peer punishment institution. Results underscore how critical it is that groups install cooperative leaders: followers model their leaders’ cooperation choices, even in decisions external to the original institution and outside of the leader’s watch.

## Introduction

Members of groups and organizations commonly face social dilemmas, or conflicts between their own interests and collective interests. From productivity on team work assignments to the mobilization of social movements, situations often arise where individuals can gain more by free-riding on the efforts of others rather than contributing to group goals. How groups motivate their members to make costly contributions for the good of the group is thus a central concern in the social sciences [1–7].

One key solution to promoting cooperation is the introduction of peer punishment, where each group member can administer punishments to the others [8–11]. While peer punishment is highly effective at deterring free-riding, it has also been associated with several critical downsides. First, peer punishment is costly and thus itself poses a social dilemma [12, 13], given that all group members would prefer that others bear the costs of administering punishment. In addition, when all can punish all, punishment can be difficult to coordinate [14], leading to over- or under-punishment. And peer punishment allows for retaliatory counter-punishment, which can result in decreased cooperation [15].

Perhaps as a result, researchers have increasingly considered an alternative solution, in which the ability to punish is given to only one group member [16–22]. Designating a single punisher can significantly reduce the costs of punishment, while avoiding the issues of coordination and retaliation. And it is more typical of real world groups, where all often do not have the ability to punish all, or at least not to the same extent [16, 17, 19, 22–24]. Indeed, groups and societies commonly grant authority figures, or leaders, with the ability to sanction defectors. These leaders are expected to contribute to the groups or societies to which they belong, but are also vested with an enhanced ability to monitor contributions and administer sanctions to their non-leader group members. For example, managers typically both contribute to their teams’ efforts on work projects, and are also granted the power to sanction poor workmanship in their subordinates via administering bonuses, conducting performance evaluations, and making decisions about promotion and termination. Given that leaders often fulfill the role of designated punishers in daily life [17], past work has thus considered whether and how group leaders, given the sole ability to sanction the members of their group, promote contributions compared to the standard solution, i.e., peer punishment [17, 19, 20, 22].

Yet research on whether the leadership approach to sanctions successfully promotes cooperation, compared to peer punishment, has yielded mixed results. One critical issue is that designated punishers are faced with incentives to abuse their power [18, 22, 25]. In groups with a designated punisher, compared to peer punishment, one individual is completely free from the risk of sanctions–the leader him or herself. And just as groups without punishment are less cooperative than groups that face the risk of sanctions [8–11], *within* groups, specific individuals who are free from punishment may be less cooperative than those who are not. The power difference between the punisher and the punished may only exacerbate these potential differences in cooperation. This issue may explain why what Carpenter and colleagues [24] refer to as “connected” punishment networks, where each member in the group can be sanctioned by at least one other individual, are generally more effective at promoting cooperation than “disconnected” networks, where one or more individuals are free from punishment. Additionally, those given the ability to punish may fail to use their power, opting to hoard their resources for themselves rather than using them to punish [22]. Generally, these issues are key downsides for the leadership solution, compared to institutions where the power to punish is distributed across all group members.

On the other hand, some other recent work has suggested that leadership is just as effective–or even more effective–than peer punishment if the leader has prosocial preferences [19, 26] or is otherwise prevented from taking advantage of his or her enhanced power, for example, by changing the designated punisher from round to round [17]. Cooperation is especially promoted when the leader is democratically elected [16, 21], a mechanism that may encourage leaders to both use the power they were granted by the electorate, and to use it responsibly. This work has demonstrated that groups with leaders given the sole ability to punish can effectively and efficiently solve social dilemmas, at least under certain conditions.

The question of whether and when the group leadership approach to sanctions will solve social dilemmas has thus been a fruitful avenue of research, as scholars seek alternatives to the standard solution in the literature—peer punishment. What this work does not yet tell us is whether, and how, leaders establish group norms that promote (or reduce) cooperation, compared to those groups with peer punishment. Nor does it tell us whether the differences in experiencing leadership vs. peer punishment systems have lasting effects *outside* of the institution. While designating a sole punisher as the group leader may solve some of the problems associated with peer punishment, it also alters the structure of the group: it establishes a hierarchy, with the leader occupying a central role [19]. Leaders are salient members of their groups, occupying an important place within them [27]. As a result, groups that opt to centralize sanctioning ability in a leader may also grant this group member with an outsize ability to set and enforce cooperative, or uncooperative, group norms.

In particular, a designated punisher may have a critical effect on group members’ behaviors, and on group outcomes on the whole, via two mechanisms: 1) the leader’s own contribution to group goals and 2) the leader’s use of his or her ability to punish. Yet past work on the leadership solution has not examined whether and to what extent leaders disproportionately affect cooperative norms in their groups via these two mechanisms, compared to peer punishment where no one individual has an enhanced role in the group. The study presented here considers, first, whether a leader’s own contributions, use of punishment, or both establish cooperative norms that promote cooperation in their non-leader group members, and how the leader’s influence fares compared to the influence of peers in a peer punishment institution.

In addition to understanding whether and how leaders’ behaviors establish cooperative norms *inside* the punishment institution, we also do not currently know whether leaders shape cooperation in their followers that lasts *outside* the institution. Recent work has shown that participating in a social dilemma under a “high quality” sanctioning system (i.e., where those who do not contribute risk punishment by the experimenter), vs. experiencing no sanctions at all, promotes cooperation even in interactions outside of the institution [28]. This work demonstrates that the sanctioning institutions one experiences is critical in fostering cooperation. But it remains unclear whether and how designated punishers’ behaviors produce high quality sanctioning systems–via their own cooperation, their use of sanctions, or both–and how their (un)cooperative choices produce lasting effects in their non-leaders’ choices via establishing cooperative norms that last, even outside of the leader’s purview.

Yet knowing whether leaders promote cooperation in their followers’ subsequent interactions, even outside of the leader’s watch, is critical. After all, in the real world, leaders are typically unable to continuously monitor their followers’ behavior. And leaders’ goals commonly involve shaping how followers will act not only in the present, but also in the future, outside of the leader’s direct observation. Generally, the institutions that govern us help shape our notions of what is right or wrong, even beyond the reach of those institutions [28]. As a result, here I also examine whether and how leaders affect their non-leader group members’ downstream cooperative behaviors, outside of the leader’s watch. Taken together, the aim of the present study is to further understand how leadership institutions fare, compared to peer punishment institutions, in fostering long-lasting cooperation.

To examine leaders’ vs. peers’ role in establishing cooperative norms via their contributions and use of sanctions, and whether the effect of these leaders’ behaviors lasts in subsequent interactions, I conducted a laboratory experiment. Four-person groups completed a standard public good dilemma, with nine rounds of a baseline non-punishment phase and nine rounds of a punishment phase. In each round of both phases, following past work [8, 9], participants were given an endowment of 20 monetary units (MUs). They simultaneously decided how many MUs, from 0 to 20, to contribute to a group fund. MUs contributed to the group fund were doubled and then distributed equally to all group members; MUs not contributed were kept for the self, but were not doubled. As a result, the task poses a social dilemma: there is a tension between what is best for the individual (to keep all resources for the self) and what is best for the group (to contribute all resources to the group fund).

In the punishment phase, either all group members (peer punishment condition) or one randomly assigned group member (leader condition) could punish the others. (The study instructions avoided the use of the term “punishment”; instead, participants were told they could “deduct points from” others). Leaders could not be punished and had their position for the remainder of the phase.

Finally, after the punishment phase, participants completed a previously unannounced one-shot version of the public good dilemma. In the one-shot task, it was emphasized that, unlike the previous task, now they and the others would not be able to see others’ contributions and earnings. Nor would anyone be able to administer or receive punishments. See the Methods section for more details and the S1 File for the full text of these instructions.

## Results

Because the data were nested in two or three levels (participants in groups for analyses on contributions in the one-shot public good dilemma, or decision rounds within participants in groups for the analyses on the repeated public good dilemma), the analyses reported here use multilevel linear regression models [29] with maximum likelihood estimation. To ensure that fixed effect estimates were robust to violations of assumptions of multilevel models (i.e., that model residuals at all levels and random coefficients at higher levels are normally distributed [29]), all reported fixed effect estimates also contain bootstrap confidence intervals using the percentile method with N = 2000 samples. Round was treated as a linear variable in order to examine general contribution trends over time. But follow-up analyses treating round as a series of dummy variables (given that round can range only from one to nine, it is not a continuous variable) did not substantively change any findings. The S1 File contains additional details and ancillary analyses.

### Preliminary analyses: group-level contributions and earnings in the public good dilemma

**Fig 1:**
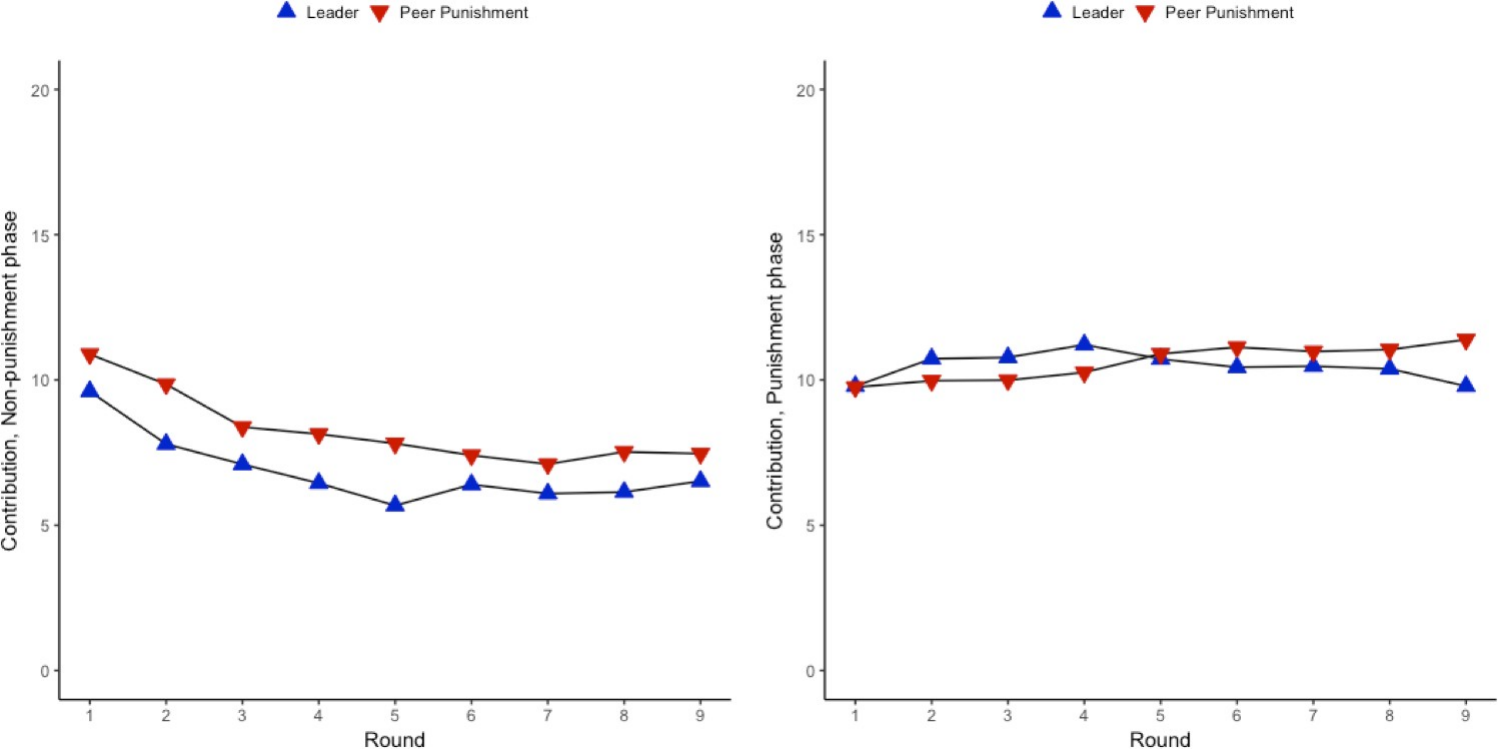
Contribution to the public good across conditions, non-punishment phase (1a) and punishment phase (1b).

Fig 1A shows group-level contributions across the nine rounds of the first phase of the study: the baseline non-punishment rounds of the public good dilemma. Preliminary analyses on cooperative behavior in this phase revealed that contributions fell across the nine non-punishment rounds (*B* = -.36, 95% bootstrap CI [-.44, -.28], *p* < .001, see Table A in S1 File, Model 1). This is the typical finding in standard public good dilemmas without punishment and demonstrates the key problem: without the presence of punishment or other incentives for cooperation, group members free-ride off of the efforts of others and contributions spiral downward dramatically. As expected, given that there was no difference in the instructions or task by condition in the non-punishment phase, there was no difference in contributions by condition (*B* = -1.42, 95% bootstrap CI [-4.19, 1.04], *p* = .28, see also Fig 1A and Table A in S1 File, Model 1) in this phase. Additionally, contributions fell over time to a similar extent in both conditions (i.e., there was not a significant round *x* condition interaction, *p* = .35, see Table A in S1 File, Model 2). Taken as a whole, these results suggest that across conditions, the groups in this phase appear statistically similar. However, because the group composition remained unchanged from the non-punishment phase to the punishment phase, behavior occurring in this phase could have influenced behavior in subsequent phases. As a result, in all subsequent analyses I control for both the individual’s contribution and the group’s average contribution in the non-punishment phase.

Between the final round of the first phase and the first round of the second phase, participants were told that sanctions would now be possible. An increase in contributions from round nine of the non-punishment phase to round one of the punishment phase would suggest that the introduction of sanctions had a deterrent effect on free-riding behavior, even before knowing how much, if any, one’s group members or the leader (depending on condition) would cooperate or punish. Analyses revealed that contributions were significantly higher in the first round of the punishment phase than in the previous round, i.e., the final round of the non-punishment phase (*B* = 2.79, 95% bootstrap CI [1.93, 3.72], *p* < .001, see Table B in S1 File, Model 1). This was the case in both conditions: the effect of introducing sanctions did not differ across conditions (i.e., the condition *x* round one of punishment phase interaction was not significant, *p* = .26, Table B in S1 File, Model 2). But note that one alternative possibility for this finding is a “restart effect”, where contributions return to higher, round one levels when the public good dilemma task is restarted, even if the rules of the task have not changed [30–32]. Thus a better test of the effect of punishment is an analysis of behavior in *all* rounds, including those later rounds where we might observe cooperation dropping over time, as in the non-punishment phase. The next analyses consider how the introduction of punishment affected contributions throughout the entirety of the phase.

Fig 1B displays the contributions across the nine rounds of the punishment phase by condition. As in the previous phase, in this phase there was no difference between the two institution types in contributions to the public good (*B* = -.08, 95% bootstrap CI [-3.34, 2.91], *p* = .96, Table C in S1 File, Model 1, see also Fig 1B). But unlike in the non-punishment phase where contributions declined dramatically, under the punishment institution, contributions remained high after their initial, significant increase in round one of the phase, staying consistently high in the leadership condition and increasing over time in the peer punishment condition (see Table C in S1 File, Model 2). That contributions remained high across the entirety of the punishment phase supports the notion that the introduction of sanctions promotes cooperation, as has been found in a host of past work [8–11]. As noted above, these results control for individual and group average contribution in the equivalent round of the non-punishment phase. Additional models containing control terms for gender, age, and pool type did not impact any of the key results; see the S1 File for these ancillary analyses).

The results on contributions appear suggestive that, overall, peer punishment and leadership were effective in promoting contributions to the public good, compared to when sanctions are not possible. But because punishments are costly, overall welfare–i.e., earnings from the public good–is determined not only by contributions, but also by the costs of punishments sent and received. And as noted above, installing one group member with the ability to administer punishment, rather than allowing all to punish all, can also keep welfare-destroying punishments low by avoiding issues of counterpunishment and coordination. Indeed, earnings after accounting for the costs of sanctions were significantly lower in the peer punishment condition, compared to the leader condition. This was the case even after controlling for contributions in the punishment phase: holding constant contributions, earnings were higher in the leader condition compared to the peer punishment condition (*B* = 7.88, 95% bootstrap CI [4.13, 11.26], *p* < .001, Table D in S1 File).

On the whole, then, these preliminary analyses suggest that leadership promotes similar contribution levels, with reduced welfare destruction via sanctions, compared to peer punishment. But these analyses take a group-level approach, and do not consider how leaders’ behaviors affect group contributions and outcomes, for the better or the worse. Nor do they consider whether and how leaders’ behavior has lasting effects on those under the leader’s watch. The next section moves from the preliminary descriptive analyses of contributions and earnings to the analyses testing the arguments above: that leader behavior in particular will establish cooperative norms in groups with leaders (compared to peer behavior in groups with peer punishment), and that these norms of cooperation will last in subsequent interactions.

### Do group leaders establish cooperative norms? The effect of leader behavior on contributions *within* the institution

As noted above, designating a solitary group member to administer sanctions alters the group facing the public good dilemma: it gives the leader enhanced power over the others, by allowing them the sole ability to administer sanctions. Did this group leader have an enhanced impact on his or her non-leader group members’ contributions, and did this impact last? Here I focus on two proposed mechanisms by which leaders may affect their followers’ behaviors and thus, group-level outcomes: the leader’s own contribution decisions and the leader’s use of his or her punishment ability. While on the whole, leadership and peer punishment may appear equally effective, those groups with leaders may have diverged significantly in their ability to provide for the public good, depending on the leader’s behavior.

To consider these differences further, Table 1 displays results from two models examining how the leader’s behavior in the previous round affected non-leaders. Model 1 predicts non-leader group members’ contribution in the next round (round *r* + 1), and contains terms for the two key leader behaviors in the previous round (round *r*): 1) the leader’s contribution decision and 2) the punishment received from the leader. The model also controls for the non-leader’s own contribution in the previous round. Because of the inclusion of terms for punishment received and leader contribution, leaders (who could not be punished) and those in the peer punishment condition (who did not have a leader) were omitted from these analyses, though I return to the peer punishment groups later.

**Table 1:** Leader contribution decision in the previous round predicts non-leader cooperation in the next round *within* the institution.

	Model 1		Model 2	
	*B (SE)*	*95% Bootstrap CI*	*B (SE)*	*95% Bootstrap CI*
Own contribution	.55 (.04)***	.48, .62	.45 (.05)***	.36, .55
Leader’s contribution	.30 (.03)***	.23, .36	.18 (.05)***	.09, .28
Prosocial punishment received	.58 (1.12)	-1.61, 2.82	.07 (1.15)	-2.17, 2.34
Antisocial punishment received	-2.15 (3.83)	-9.71, 5.31	-3.89 (3.89)	-11.31, 3.86
Group average contribution			.29 (.08)***	.12, .45
Own contribution in the non-punishment phase	.07 (.03)*	.01, .13	.07 (.03)*	.01, .14
Group average contribution in the non-punishment phase	.04 (.05)	-.06, .13	.00 (.05)	-.09, .09
Round	.05 (.06)	-.07, .18	.00 (.06)	-.12, .13
**Intercept**	.39 (.50)	-.62, 1.40	.20 (.44)	-.67, 1.05
**Variance Components**				
Level 1	13.00 (3.61)		12.91 (3.59)	
Level 2	.00 (.00)		.33 (.57)	
Level 3	.50 (.71)		.00 (.00)	

**Note:** + *p* < .10

* *p* < .05, ** *p* < .01

*** *p* < .001.

Multilevel linear regression model (maximum likelihood estimation) with random intercepts for participant and group. All variables in the model denote behavior in the current round (round *r*); the dependent variable is contribution in the next round (round *r* + 1). This model contains groups in the leader condition only. The peer punishment condition, the leaders, and round nine behavior (because behavior in the next round does not exist in round nine) are omitted from analyses. N = 672 rounds (nested in 84 participants nested in 28 groups). Contribution variables range from 0 to 20; punishment received variables range from 0 to 1.

Results revealed that, holding constant the leader’s use of punishment (which I discuss in turn), the group leader’s own contribution decision in a given round is significantly predictive of non-leader contributions in the subsequent round (*B* = .30, 95% bootstrap CI [.23, .36], *p* < .001, Table 1, Model 1). Designated punishers’ own cooperative behavior is a critical key in determining whether their groups will succeed or fail at producing the public good, even holding constant their use of punishment. Highly cooperative group leaders induce cooperation in their followers in subsequent decisions; those who cooperate less are less likely to encourage cooperation among their non-leader counterparts. Additional control terms for gender, age, and pool type did not affect results; see Table E in S1 File.

Next I turn to the punishment received from the leader. Given that punishment is proposed to be more likely to promote cooperation when it is administered by high contributors to low contributors than when it is antisocial, i.e., administered by low contributors to high contributors [33–35], I computed two separate variables for punishment received: the amount of prosocial punishment received (i.e., punishment directed by a group member—here, a leader–who had contributed more than the recipient in the round) and the amount of antisocial punishment received (i.e., directed by a group member who had contributed as much as or less than the recipient), following past work [33]. To facilitate comparisons with the peer punishment condition in analyses described later (given that the maximum punishment that one could receive differed in the leader condition vs. peer punishment condition), the punishment received variables were standardized as the proportion of punishment received out of the maximum possible punishment one could receive (10 MUs in the leader condition, 30 MUs in the peer punishment condition– 10 MUs from each of three group members). As a result, the standardized punishment received variables could range from 0 (e.g., the participant received no prosocial punishment in this round) to 1 (e.g., the participant received the maximum amount of prosocial punishment possible in this round).

As shown in Table 1, punishment received from the leader in the previous round, whether it was prosocial or antisocial in nature, did not significantly predict increased contributions in the next round, holding constant the leader’s contribution in the previous round (*p* = .60 and *p* = .57 respectively, Table 1, Model 1). Of course, this does not imply that sanctions do not promote cooperation. First, because half of the groups were omitted from these analyses, power is low. Later analyses include the peer punishment groups and allow for enhanced power to detect changes in cooperation after receiving punishment. Perhaps more importantly, the results on changes in contributions from the final round of the non-punishment phase to the first round of the punishment phase show that sanctions served as an important *deterrent*, promoting cooperation even in round one and throughout the remainder of the phase. That is, the *capacity* to sanction, whether or not sanctions were actually deployed, promoted cooperation. But it is also clear that leader contributions are critical in shaping subsequent cooperative behavior in the groups to which they belong, beyond simply introducing the ability for a leader to punish.

One explanation for these findings is not that the leader’s contribution decision in the previous round promoted non-leaders’ cooperation in the subsequent round, but that the contributions made by *all* group members, both the leader and other non-leaders alike, were weighted equally when a given group member decided how they ought to contribute in the next round. Rather than being a particularly salient group member, the leader’s behavior may have been factored into decision-making just as any other group member’s behavior. After all, people generally conform to the cooperative behavior of their group members [36, 37]. In order to demonstrate whether the findings support the argument above–that because the designated punisher occupies a more salient position in the group, the leader’s contribution in *particular* will determine his or her group members’ actions, Model 2 includes a term for the group’s average contribution on the whole.

Perhaps unsurprisingly, group-level cooperative behavior in the previous round predicted own cooperative behavior in the next round. This suggests that there was a “baseline influence” effect, where people took into account all of their group members’ previous contributions in general when deciding how much to cooperate in the current decision. However, the effect of the leader’s contribution behavior in *particular* remained significant, even after controlling for this baseline influence effect (*B* = .18, 95% bootstrap CI [.09, .28], *p* < .001, Table 1, Model 2). Leaders were especially salient in promoting (or reducing) cooperative decisions among their group members. Their choices made a larger impact than did the choices of their non-leader counterparts.

But note that the above analyses necessitated omitting the peer punishment condition, because of the inclusion of a term for the leader’s contribution decision. It is possible that peer punishment institutions are equally effective (poor) at establishing cooperative norms, compared to those with leaders, if they have a high (low) contributor. That is, one possibility for the similar overall cooperation levels between leader groups and peer punishment groups is that leader groups can yield either high or low contribution levels, depending on the leader’s behaviors, while peer punishment groups, less able to establish a norm around one salient group member, fall somewhere in the middle. But another possibility is that peer punishment groups are also just as influenced by the other group members and also yield very high or very low contributions, depending on the presence of a particularly high or particularly low contributor.

As a result of these issues, in follow-up analyses I matched each peer punishment group with a corresponding, unique leader group. (There was one more group in the leader condition than in the peer punishment condition, so all peer punishment groups were successfully matched with a unique leader group.) I designated a “selected group member” in each peer punishment group as the group member whose ID corresponded to the ID of the leader in the paired leader group (e.g., if the leader was participant A, the selected group member in the corresponding peer punishment group was participant A, and so on). This yielded a randomly chosen group member whose influence over the other members of the group could be compared to that of the influence of the leader observed in the leader groups.

Table 2 displays results of models like those in Table 1, but with the peer punishment condition included. Model 1 considers whether contributions were impacted by the behavior of a selected member of the group in the previous round–that selected member being the leader in the leader condition, or a randomly chosen peer (held constant across rounds) in the peer punishment condition. Perhaps unsurprisingly given the results described above, across conditions, this selected group member’s behavior did influence cooperation among his or her fellow group members in the subsequent round (*B* = .19, 95% bootstrap CI [.15, .23], *p* < .001). This occurred even after controlling for own behavior in the previous round, as well as punishment received in the previous round, which (unlike the analyses within the leader condition alone) was also associated with cooperation, if it was prosocial in nature (*B* = 2.88, 95% bootstrap CI [1.18, 4.61], *p* < .001).

**Table 2:** Leaders’ cooperation is more influential in promoting group members’ cooperation in the next round, compared to peers’ behaviors in peer punishment institutions.

	Model 1		Model 2		Model 3	
	*B (SE)*	*95% Bootstrap CI*	*B (SE)*	*95% Bootstrap CI*	*B (SE)*	*95% Bootstrap CI*
Own contribution	.70 (.02)***	.66, .74	.69 (.02)***	.65, .74	.69 (.02)***	.65, .73
Selected group member’s contribution	.19 (.02)***	.15, .23	.16 (.03)***	.11, .22	.16 (.03)***	.11, .21
Leader condition			-.98 (.46)*	-1.88, .02	-1.05 (.48)*	-1.98, .06
Leader condition x Selected group member’s contribution			.07 (.03)*	-.00, .14	.07 (.03)+	-.00, .14
Prosocial punishment received	2.88 (.86)***	1.18, 4.61	2.71 (.87)**	1.00, 4.47	1.74 (1.49)	-1.13, 4.80
Antisocial punishment received	-.08 (1.58)	-3.17, 3.20	-.38 (1.61)	-3.48, 2.84	-.66 (1.81)	-4.26, 2.88
Prosocial punishment received x Leader condition					1.30 (1.68)	-2.07, 4.57
Antisocial punishment received x Leader condition					.71 (4.16)	-7.45, 8.98
Own contribution in the non-punishment phase	.10 .02***	.06, .14	.10 (.02)***	.06, .15	.10 (.02)***	.06, .14
Group average contribution in the non-punishment phase	-.03 (.03)	-.09, .04	-.03 (.03)	-.10, .04	-.03 (.03)	-.10, .04
Round	.04 (.04)	-.05, .13	.05 (.04)	-.04, .13	.05 (.04)	-.04, .14
**Intercept**	.33 (.36)	-.38, 1.01	.82 (.43)+	-.05, 1.63	.89 (.44)*	-.01, 1.73
**Variance Components**						
Level 1	12.48 (3.53)		12.44 (3.53)		12.42 (3.52)	
Level 2	.00 (.00)		.00 (.00)		.00 (.00)	
Level 3	.54 (.74)		.53 (.73)		.56 (.75)	

**Note:** + *p* < .10

* *p* < .05

** *p* < .01

*** *p* < .001.

Multilevel linear regression model (maximum likelihood estimation) with random intercepts for participant and group. All variables in the model denote behavior in the current round (round *r*); the dependent variable is contribution in the next round (round *r* + 1). The “selected group member” is either the leader (in the leader condition) or a randomly chosen peer, held constant across rounds (peer punishment condition). The selected group members themselves, as well as round nine behavior (because behavior in the next round does not exist in round nine) are omitted from analyses. N = 1320 rounds (nested in 165 participants nested in 55 groups). Contribution variables range from 0 to 20; punishment received variables from 0 to 1.

**Fig 2:**
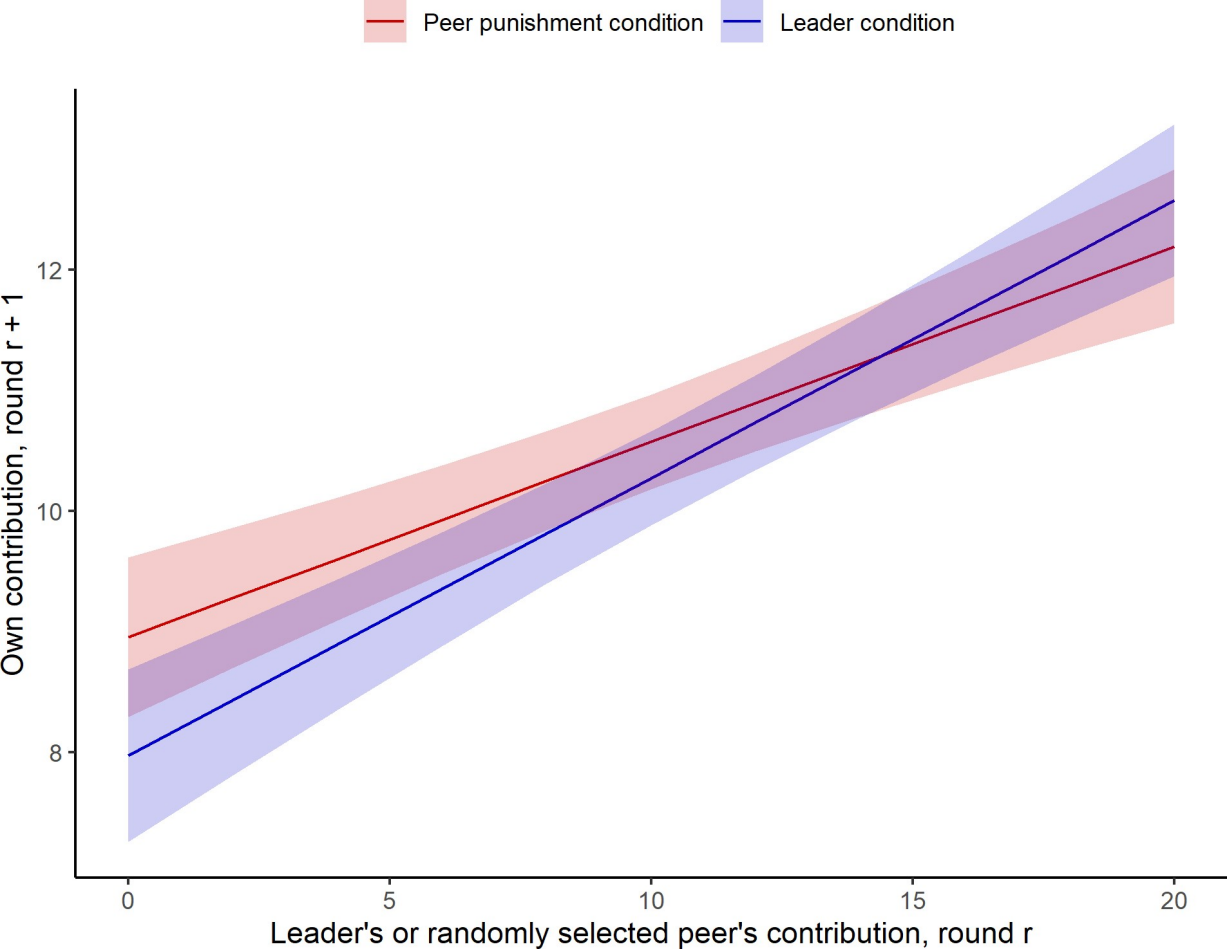
The effect of the leader’s contribution, vs. a peer’s contribution, on promoting cooperation among the other members of the group. **Note**: Fig 2 displays estimates for contribution as predicted from Model 2, with the remaining predictors held constant at their means.

But were leaders *more* influential than their counterparts in the peer punishment condition at establishing norms of cooperation? Model 2 demonstrates that they were. The effect of the selected group member’s behavior is stronger in the leader condition, compared to the peer punishment condition, as demonstrated by a significant interaction between the selected group member’s behavior and condition. The main effects are also significant and can be interpreted as follows. In the peer punishment condition, the selected group member’s behavior influences his or her group members’ contributions in the next round (main effect of the selected group member’s contribution, *B* = .16, 95% bootstrap CI [.11, .22], *p* < .001). When the selected group member gave 0 in the previous round, giving in the next round was significantly lower in the leader condition, compared to in the peer punishment condition (main effect of condition, *B* = -.98, 95% bootstrap CI [-1.88, -.02], *p* = .04), suggesting that leaders, compared to peers, are more likely to reduce others’ cooperation when they themselves give nothing. Finally, the selected group member’s contribution was more impactful on others’ contributions in the leader condition (i.e., when the selected group member was the leader), compared to in the peer punishment condition (selected group member’s contribution *x* leader condition interaction, *B* = .07, 95% bootstrap [-.00, .14], *p* = .05). Fig 2 illustrates the effect of selected group members’ contributions on their fellow group members’ contributions in the next round by condition, as predicted from Model 2. A high contributing leader is more effective than a high contributing peer at establishing high norms of cooperation in the group. Likewise, a low contributing leader is more detrimental to the group, inducing followers to contribute less.

Finally, I also examined whether the effect of receiving punishment differed across conditions. Model 3 demonstrates that the interaction between antisocial or prosocial punishment received and condition was not significant (*p* = .86 and *p* = .44, respectively). Rather, in this study, the introduction of sanctions in round one and then their use throughout the punishment phase appeared to affect participants similarly, regardless of whether the punishment was administered by a peer or a leader. On the other hand, the effect of others’ *contributions* is particularly high in groups with leaders–leaders’ cooperative choices establish norms that ultimately help or harm their groups.

The second key question of interest was whether leaders’ behavior has a lasting effect on followers even *outside* of the institution and the leader’s purview. The next section considers contribution behavior in the one-shot public good dilemma, once participants were told that the institution was removed and punishment was no longer possible.

### Do cooperation differences last? The effects of leader behavior on contributions *outside* the institution

**Fig 3:**
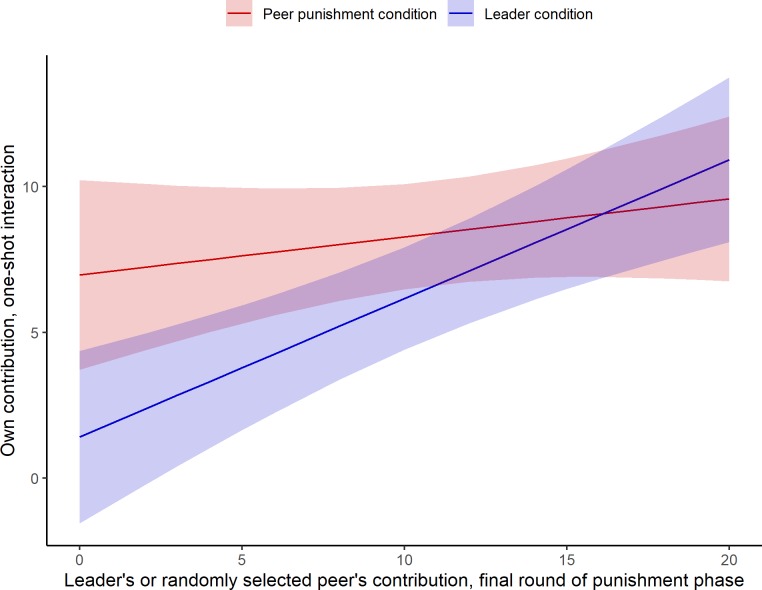
The effect of the leader’s contribution, vs. a peer’s contribution, on promoting cooperation in a subsequent, anonymous one-shot interaction. **Note**: Fig 3 displays estimates for contributions in the one-shot dilemma, as predicted from the model given in the paragraph above.

The results described above demonstrate that, across the leader and peer punishment conditions, leaders’ contribution behavior (vs. that of a given peer’s contribution behavior) played an especially important role in establishing group cooperative norms. Similarly, a model containing terms for the leader’s or randomly selected peer’s contribution in the final round of the previous phase, condition, and their interaction revealed that, again, the leader’s cooperative behavior was significantly more influential in promoting cooperation among the others, even in the one-shot interaction when the institution was no longer in place and punishment was not possible. Specifically, in the peer punishment condition, the peer’s giving behavior in the previous round did not predict one-shot giving (the main effect of the selected group member’s contribution was not significant, *B* = .13, 95% bootstrap CI [-.12, .39], *p* = .30). When leaders gave 0, their followers gave significantly less in the one-shot dilemma than those whose peer had given nothing (main effect of the leader condition, *B* = -5.57, 95% bootstrap CI [-9.89, -1.29], *p* = .02). And the more the leader gave, the more his or her group members gave in the subsequent one-shot dilemma (selected group member’s contribution *x* leader condition interaction, *B* = .35, 95% bootstrap CI [.01, .68], *p* = .05). Fig 3 illustrates the effect of selected group members’ contributions in the final round of the punishment phase on their fellow group members’ contributions in the anonymous, one-shot dilemma, as predicted from this model. Again, high contributing leaders are more effective–and low contributing leaders are less effective–than high or low contributing peers at establishing cooperation that lasts, shaping others’ behavior in subsequent interactions where behavior is anonymous and punishment is not possible.

Given that leaders’ behaviors were again demonstrated as being more influential than that of peers, in follow-up analyses I restricted analysis to the leader condition, as in Table 1 above, to conduct a mediation analyses on whether leaders’ influence on their followers’ cooperative or uncooperative behavior *within* the institution mediated their continuing influence *outside* of the institution. After all, when making cooperative choices outside the institution, people may have simply opted to do what was working well for them in the past–that is, what they did inside the institution. This does not diminish the importance of leaders’ behaviors in promoting follower cooperation. Instead, it suggests that leaders’ promoting cooperation in their followers at *earlier* stages–in the institution–may yield differing patterns of behavior that persist even *outside* the institution. Leaders’ behavior is thus critical in establishing initial cooperative choices in others.

A model with predictors for the leader’s total contributions across the nine rounds of the punishment phase and the total punishment received from the leader across the nine rounds allows for examination of whether either of these leader behaviors predicted their followers’ cooperation in the one-shot PDG—after the institution, i.e., the possibility for punishment, was removed. Results revealed that the more the leader had contributed in the punishment phase of the public good dilemma, the more non-leaders contributed in the subsequent one-shot dilemma (*B* = .06, 95% bootstrap CI [.03, .09], *p* < .001). These results also control for total prosocial and antisocial punishment received from the leader, which were not significantly associated with one-shot giving. Consistent with the findings from the round-level analyses described above (see Table 1), a model regressing total leader giving on the mediator (i.e., non-leader giving within the institution) revealed that total leader giving significantly predicted non-leaders’ own contributions within the institution (*B* = .84, 95% bootstrap CI [.71, .97], *p* < .001). And, once non-leaders’ own giving within the institution was included in the model, the effect of leader giving on cooperation in the one-shot PDG (i.e., cooperation outside the institution) became non-significant (*p* = .39).

**Fig 4:**
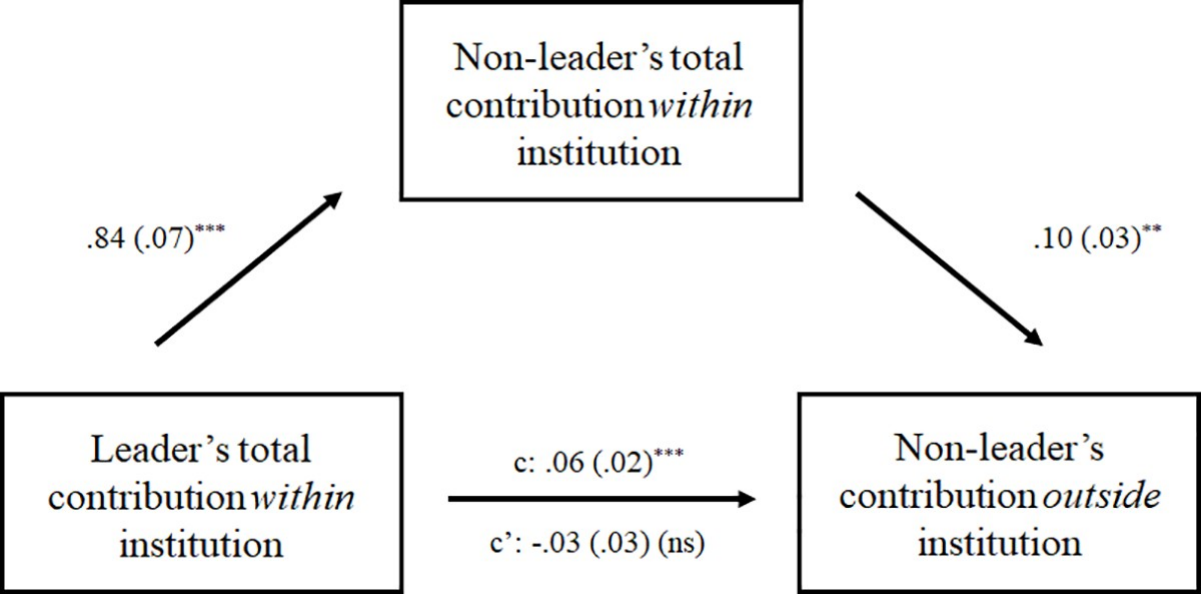
Mediation analyses, relationship between leader cooperative behavior within the institution and non-leader cooperation outside of the institution. **Note:** + *p* < .10, * *p* < .05, ** *p* < .01, *** *p* < .001. Mediation analyses consisting of multilevel linear regression models (maximum likelihood estimation) with random intercepts for the group. Models also contained control terms for prosocial and antisocial punishments received within the institution. N = 84 participants nested in 28 groups. See also Table F in S1 File.

That is, leader behavior in the institution promoted cooperative behavior outside of the institution, via an increase in followers’ behavior inside the institution. Leader cooperation shapes followers’ cooperation, which lasts even when the leader cannot sanction and the interaction is completely anonymous and free from punishment. Importantly, as shown above, it does so above and beyond the influence of peers in peer punishment institutions. Fig 4 displays the results of the mediation analysis. The full models for these analyses are also displayed in Table F in S1 File.

## Discussion

While much past work has demonstrated that the introduction of peer punishment promotes cooperative behavior, as noted above, peer punishment faces several downsides. Designating a single group member to administer punishment addresses several of these key downsides, but as I show here, it also gives the leader disproportionate ability to shape group norms. This study demonstrates that, on the whole, leadership can promote cooperation to a similar extent as peer punishment. However, a deeper examination into the effect of leaders’ behaviors on group-level outcomes reveals that leadership will be an effective solution *if* the leader engages in cooperative behavior when he or she obtains the role. Indeed, the leader’s own cooperation behavior is more important than peers’ cooperation behavior in peer punishment institutions. If leaders engage in cooperative behavior, their groups can achieve larger public good production than those in peer punishment institutions, while avoiding the downsides of peer punishment, including coordination problems and high costs. At the same time, leadership will fail if the leader opts not to cooperate—in these situations, peer punishment is more likely to yield successful public good production.

Still further, and perhaps more importantly, the outsize effect of leaders’ cooperative behavior on promoting followers’ cooperation lasts even when the institution is removed. Cooperative leaders establish cooperative group norms that shape followers’ decisions in subsequent interactions. That leaders who model cooperation for their followers promote follower cooperative behavior even in those followers’ subsequent interactions is a previously unexamined benefit of cooperative leadership.

Of course, in this study the group member in the leader position was endowed with a special ability within the group: the ability to punish. While the key results tended to center on leaders’ contribution behaviors, this is not to say that the leader’s use of punishment is not important. As noted earlier, past work has suggested that granting punishment ability to one group member will fail if that group member chooses to hoard resources rather than using them to punish [22]. Taken together with the results discussed here, leaders who both cooperate and are willing to punish noncooperation–i.e., those who are “strong reciprocators” [38–40]–are likely to be most effective at promoting the public good. That is, this work builds on work suggesting that punishment is important in promoting cooperation by suggesting that not only should group leaders be willing to use their resources to sanction group members, they must also be willing to contribute to the group, modelling cooperative choices rather than simply enforcing them in others. In doing so, they will influence the members of their groups above and beyond that of peers, and even in the choices they make outside of the leader’s watch.

To successfully promote collective action, then, it is imperative that people install the “right” leaders. While the “right” leaders, based on the discussion above, are likely to be those that are willing to cooperate and punish noncooperation in others, other types of leader behaviors are likely to be important as well. Because this study was conducted in anonymous four-person groups, for example, demographic characteristics were not as able to play a role as they might in real-world, face to face interactions. For instance, some past work suggests that high cooperators who are high status are more likely to promote others’ cooperation, compared to high cooperators who are low status [41]. Additionally, past work has demonstrated that leaders with a prosocial social value orientation are more likely to promote cooperation than those who are proself [19]. This work builds upon this previous research by showing that it is not necessary for leaders to be categorized as a prosocial “type” to promote cooperation in their groups, so long as they engage in cooperative behavior. As a result, even those leaders who are proself “types”, if they are induced to cooperate (due to the presence of reputational benefits [42], to provide one example), are likely to promote cooperative norms that last. On the whole, whether the effects of the study here are moderated by these and other leader traits or characteristics could be tested via extensions of the experiment described above.

Note that, like any study, this one has limitations. One issue is that, in this experiment, the maximum amount of punishment that one could have received in the leader condition differed from that of the peer punishment condition (10 MUs in the leader condition; 10 from each of three others, thus 30 MUs total, in peer punishment). As a result, one unit of punishment may have carried greater weight in the leader condition, resulting in differing levels of punishment use and welfare destruction due to the study design. Note that this issue does not directly bear on the key results in this work, that is, on leaders’ contribution behaviors promoting cooperative behaviors in their followers. Nevertheless, future work might, for example, distribute punishment “points” across group members or centralize them in one group member, holding constant the total number of these points available in the group. Such a study could answer additional questions about the effects of the distribution of punishment power on cooperation that this one was not designed to address.

Further, this study relied on the same behavioral outcome both within and outside the institution: contribution to a public good with the members of one’s group. The key difference was that the decision outside the institution was one-shot, not relayed to the group members, and free from sanctions. By relying on a highly similar behavior that participants had experienced over the course of several rounds, it is possible that part of their decision in the interaction outside of the institution was due to inertia: sticking with the same strategy they used previously. Granted, the results stand: those with cooperative leaders behaved more cooperatively while those with cooperative peers were less affected; this enhanced cooperation predicted further cooperation even when sanctions were removed. Nevertheless, future work might consider whether the effect of cooperative leadership “spills over” into subsequent interactions that are more distinct from the interactions within the institution (for instance, a measure of generosity like the dictator game, as in some prior related work [28]). This would yield additional insights into whether and in what contexts cooperative leadership promotes cooperative followers that are beyond the scope of the current work.

Additional considerations for future work might consider the varying ways that leadership is conceptualized in research on groups facing social dilemmas. While, as noted above, past work has examined designated punishers as authority figures or group leaders [16–21], others have considered different forms of leadership in these groups. Most notably is the “leading-by-example” paradigm in which leaders make contributions first, then, the remaining group members make their own decisions [43–46]. It is thus far unclear whether the effect of a leader who contributes first sets an example that persists in *subsequent* interactions, once the leader is no longer present. In addition, past work considering leading-by-example has found that while followers’ contributions are strongly influenced by the leader’s contribution, leaders are often exploited by followers, reducing the willingness to engage in costly leadership; generally, results as to whether leading by example works are mixed [43–46]. Future work might consider whether some combination of “leading-by-example” and leader ability to sanction may be particularly effective at promoting cooperation. Leaders who both cooperate first and are able to sanction may harness the benefits of both forms of leadership, and as in the study presented here, continue to influence their followers outside of the institution.

In summary, the threat of free-riding on others’ contributions to group goals makes the marshalling of cooperation a critical challenge. Solving the cooperation problem is of paramount importance to promoting productive and harmonious interactions between individuals, groups, and nations. After all, groups, organizations, and the world on the whole face this challenge when tackling major societal issues, including maintaining clean air and water supplies, promoting blood donation, and combating climate change. Notably, we commonly install leaders, given the ability to monitor and enforce cooperation, for the purpose of solving these problems. The results from this study demonstrate how important it is that groups make efforts to promote cooperative leadership. Further, they offer a more thorough understanding of the important role leaders play in inducing individuals to forgo their self-interest for the greater good.

## Methods

Participants were recruited from the general student population at the University of Michigan, via two non-overlapping participant pools–one for payment and one for course credit plus the chance of payment (see the S1 File for more details and for additional analyses demonstrating that behavior and results did not differ based on pool type). Upon arrival to the laboratory, they were escorted to a private room where they completed the study entirely via the computer. The study was programmed in z-Tree version 3.4.2 [47]. Sessions were scheduled in four-person groups and each group was randomly assigned by the computer program to either the peer punishment (N = 108 participants in 27 groups) or leader (N = 112 participants in 28 groups) condition.

After a consent screen and several demographic items, participants completed a standard public good dilemma (PGD). Specifically, in each of a series of rounds, each group member received an endowment of 20 monetary units (MUs). They independently decided how many of their own MUs, from 0 to 20, to contribute to a “group fund”. Points not contributed to the group fund were kept in a “personal fund”. Contributions to the group fund (but not those kept in the personal fund) were doubled and redistributed equally among all group members, regardless of each person’s contributions. Thus, withholding contributions maximizes an individual’s earnings, but overall group earnings are maximized when all group members contribute. The full text of the instructions is available in the S1 File.

In the first phase of the PGD, after making their contribution decisions, participants could view their own and others’ contributions and earnings, sorted by each group member’s unique letter identifier. Then, they proceeded to the next round. This non-punishment phase was repeated for nine rounds in total and measures baseline cooperation before the punishment phase was introduced. To avoid end-game effects, participants were not told in advance how many rounds they would complete.

After the non-punishment phase was completed, additional instructions informed the participants that they would continue making similar decisions with the same three other group members, but some of the rules of the task would change. Now, following each round of the task, either “one group ‘leader’” (leader condition) or “each group member” (peer punishment condition) would, after viewing contribution decisions, be able to deduct points from the others. Note that by referring to the leader explicitly as “the group leader” in the study instructions (rather than a more generic term like “designated punisher”) the condition is likely to have activated in participants’ minds the role of leaders, and status-differentiated hierarchies of leader and followers, in a way that the peer punishment condition (where everyone was simply referred to as “group members”) did not. At the same time, by calling the group leaders what they were—“leaders” given the sole ability to sanction others—the study simulates real world conditions where leaders’ position, title, and enhanced abilities are explicit to leaders and followers alike. It is also consistent with prior work [18, 21].

Following past work, punishment was costly: it cost one MU to deduct 3 MUs from another participant [8, 9, 18, 19, 21]. Those with punishment ability (i.e., all group members in the peer punishment condition and the leader in the leader condition) could spend up to 10 of their own MUs to deduct up to 30 MUs from the others’ earnings. This meant that in the leader condition, the maximum total punishments a group member could receive was 10 MUs from the leader (resulting in a 30 point loss); in the peer punishment condition, up to 30 MUs (10 MUs from each of three other group members, resulting in a 90 point loss).

Those in the leader condition were told that the leader could not be punished, and he or she would have the position for the remainder of the study. They were also told, correctly, that the leader would be randomly selected by the computer program, and were informed whether or not they had been selected to be the leader before beginning the first round of the punishment phase. In both conditions, the instructions further stated that participant identifiers would change in this next phase. This prevented participants from deploying punishments based on the target’s behavior in the previous (i.e., the non-punishment) phase. The full text of these instructions is reproduced in the S1 File.

Throughout the punishment phase, after simultaneously making contribution decisions, all group members viewed the results (contributions and earnings) before punishment. Then, those who could administer punishments made their deduction decisions. Finally, all group members could see the results after accounting for deductions, including, in the peer punishment condition, who punished whom. This phase was repeated for another nine rounds.

After the punishment phase, participants completed a previously unannounced, one-shot PGD; again, this third phase was completed with the same three other group members. Specifically, instructions indicated that all group members would receive one final endowment of 20 MUs and again decide how many, from 0 to 20, to contribute to the group fund. However, unlike the previous task, they would make this decision only once and they would not receive any feedback on their own or others’ contributions or earnings, as they had in previous phases. Likewise, since they could not see others’ decisions or earnings, they and the others (or the leader, depending on condition) would not have the opportunity to make deductions in this round. That is, the instructions clearly emphasized that 1) this decision was fully private and 2) the punishment institution (whether peer punishment or leadership) was no longer in place. See the S1 File for more.

Upon completion of the study, participants were debriefed and paid. The study took about 45 minutes. There was no deception. The study procedures were approved by the University of Michigan’s Health Sciences and Behavioral Sciences Division Institutional Review Board (HUM00159425 for pool 1 and HUM00152808 for pool 2).

## Supporting information

S1 File Supporting information. File contents include the full text of study instructions, data and analytic strategy supplementary details, and supplementary models.
(PDF)

## References

[1] Kollock P. Social dilemmas: The anatomy of cooperation. Annu Rev Sociol. 1998;24: 183–214.

[2] Nowak MA. Five rules for the evolution of cooperation. Science. 2006;314: 1560–3. 10.1126/science.1133755 17158317 PMC3279745

[3] Rand DG, Nowak MA. Human cooperation. Trends Cogn Sci. 2013;17: 413–25. 10.1016/j.tics.2013.06.003 23856025

[4] Simpson B, Willer R. Beyond altruism: Sociological foundations of cooperation and prosocial behavior. Annu Rev Sociol. 2015;41: 43–63.

[5] Olson M. The logic of collective action: Harvard University Press; 2009.

[6] Ostrom E. Collective action and the evolution of social norms. J Econ Perspect. 2000;14: 137–58.

[7] Henrich J. Cooperation, punishment, and the evolution of human institutions. Science. 2006;312: 60–1. 10.1126/science.1126398 16601179

[8] Fehr E, Gachter S. Cooperation and punishment in public goods experiments. Am Econ Rev. 2000;90: 980–94.

[9] Fehr E, Gächter S. Altruistic punishment in humans. Nature. 2002;415: 137 10.1038/415137a 11805825

[10] Shinada M, Yamagishi T. Punishing free riders: direct and indirect promotion of cooperation. Evolution and Human Behavior. 2007;28: 330–9.

[11] Boyd R, Gintis H, Bowles S, Richerson PJ. The evolution of altruistic punishment. Proc Natl Acad Sci USA. 2003;100: 3531–5. 10.1073/pnas.0630443100 12631700 PMC152327

[12] Heckathorn DD. Collective action and the second-order free-rider problem. Rationality and society. 1989;1: 78–100.

[13] Oliver P. Rewards and punishments as selective incentives for collective action: theoretical investigations. Am J Sociol. 1980;85: 1356–75.

[14] Diekmann A, Przepiorka W. Punitive preferences, monetary incentives and tacit coordination in the punishment of defectors promote cooperation in humans. Sci Rep. 2015;5: 10321 10.1038/srep10321 25988875 PMC4437292

[15] Nikiforakis N. Punishment and counter-punishment in public good games: Can we really govern ourselves? J Pub Econ. 2008;92: 91–112.

[16] Baldassarri D, Grossman G. Centralized sanctioning and legitimate authority promote cooperation in humans. Proc Natl Acad Sci USA. 2011;108: 11023–7. 10.1073/pnas.1105456108 21690401 PMC3131358

[17] O'Gorman R, Henrich J, Van Vugt M. Constraining free riding in public goods games: designated solitary punishers can sustain human cooperation. Proc R Soc Lond B Biol Sci. 2008;276: 323–9. 10.1098/rspb.2008.1082 PMC2674351 18812292

[18] Kuwabara K, Yu S. Costly punishment increases prosocial punishment by designated punishers: Power and legitimacy in public goods games. Soc Psychol Q. 2017;80: 174–93.

[19] Harrell A, Simpson B. The dynamics of prosocial leadership: Power and influence in collective action groups. Soc Forces. 2015;94: 1283–308.

[20] Kosfeld M, Rustagi D. Leader punishment and cooperation in groups: Experimental field evidence from commons management in Ethiopia. Am Econ Rev. 2015;105: 747–83.

[21] Harrell A. Competition for leadership promotes contributions to collective action. Soc Forces. 2018;97: 405–26.

[22] Nosenzo D, Sefton M. Promoting cooperation: the distribution of reward and punishment power In: Van Lange PAM, Rockenbach B, Yamagishi T, editors. Social dilemmas: New perspectives on reward and punishment. Oxford University Press; 2014.

[23] Guala F. Reciprocity: Weak or strong? What punishment experiments do (and do not) demonstrate. Behav Brain Sci. 2012;35: 1–15. 10.1017/S0140525X11000069 22289303

[24] Carpenter J, Kariv S, Schotter A. Network architecture, cooperation and punishment in public good experiments. Review of Economic Design. 2012;16: 93–118.

[25] Van der Heijden E, Potters J, Sefton M. Hierarchy and opportunism in teams. J Econ Behav Organ. 2009;69: 39–50.

[26] Gächter S, Nosenzo D, Renner E, Sefton M. Who makes a good leader? Cooperativeness, optimism, and leading‐by‐example. Econ Inq. 2012;50: 953–67.

[27] Ahlquist JS, Levi M. Leadership: What it means, what it does, and what we want to know about it. Annu Rev Polit Sci. 2011;14: 1–24.

[28] Stagnaro MN, Arechar AA, Rand DG. From good institutions to generous citizens: Top-down incentives to cooperate promote subsequent prosociality but not norm enforcement. Cognition. 2017;167: 212–54. 10.1016/j.cognition.2017.01.017 28249658 PMC5875418

[29] Snijders TA, Bosker RJ. Multilevel analysis: an introduction to basic and advanced multilevel modeling: Sage; 1999.

[30] Andreoni J. Why free ride? Strategies and learning in public goods experiments. 1988;37: 291–304.

[31] Croson RTA. Partners and strangers revisited. Econ Letters. 1996;53: 25–32.

[32] Norton DA. Killing the (coordination) moment: How ambiguity eliminates the restart effect in voluntary contribution mechanism experiments. 2015;126: 1–5.

[33] Herrmann B, Thöni C, Gächter S. Antisocial punishment across societies. Science. 2008;319: 1362–7. 10.1126/science.1153808 18323447

[34] Rand DG, Armao IV JJ, Nakamaru M, Ohtsuki H. Anti-social punishment can prevent the co-evolution of punishment and cooperation. J Theor Biol. 2010;265: 624–32. 10.1016/j.jtbi.2010.06.010 20540952 PMC3290516

[35] Fu T, Putterman L. When is punishment harmful to cooperation? A note on antisocial and perverse punishment. J Econ Sci Assoc. 2018;4: 151–64.

[36] Wu J-J, Li C, Zhang B-Y, Cressman R, Tao Y. The role of institutional incentives and the exemplar in promoting cooperation. Sci Rep. 2014;4: 6421 10.1038/srep06421 25242265 PMC5377375

[37] Suri S, Watts DJ. Cooperation and contagion in web-based, networked public goods experiments. PloS ONE. 2011;6: e16836 10.1371/journal.pone.0016836 21412431 PMC3055889

[38] Bowles S, Gintis H. The evolution of strong reciprocity: cooperation in heterogeneous populations. Theor Pop Biol. 2004;65: 17–28. 14642341 10.1016/j.tpb.2003.07.001

[39] Fehr E, Fischbacher U, Gächter S. Strong reciprocity, human cooperation, and the enforcement of social norms. Hum Nat. 2002;13: 1–25. 10.1007/s12110-002-1012-7 26192593

[40] Gintis H. Strong reciprocity and human sociality. J Theor Biol. 2000;206: 169–79. 10.1006/jtbi.2000.2111 10966755

[41] Weber JM, Murnighan JK. Suckers or saviors? Consistent contributors in social dilemmas. J Pers Soc Psychol. 2008;95: 1340 10.1037/a0012454 19025287

[42] Simpson B, Willer R. Altruism and indirect reciprocity: The interaction of person and situation in prosocial behavior. Soc Psychol Q. 2008;71: 37–52.

[43] Drouvelis M, Nosenzo D. Group identity and leading-by-example. J Econ Psychol. 2013;39: 414–25.

[44] Moxnes E, Van der Heijden E. The effect of leadership in a public bad experiment. J Conflict Resolut. 2003;47: 773–95.

[45] Potters J, Sefton M, Vesterlund L. Leading-by-example and signaling in voluntary contribution games: an experimental study. Economic Theory. 2007;33: 169–82.

[46] Levati MV, Sutter M, Van der Heijden E. Leading by example in a public goods experiment with heterogeneity and incomplete information. J Conflict Resolut. 2007;51: 793–818.

[47] Fischbacher U. z-Tree: Zurich toolbox for ready-made economic experiments. Exp Econ. 2007;10(2): 171–8.

