# Supplementary material for: Group leaders establish cooperative norms that persist in subsequent interactions
Source: PLoS One. 2019 Sep 19;14(9):e0222724. doi: 10.1371/journal.pone.0222724 (PMC6752774; doi:10.1371/journal.pone.0222724)
Supplement: S1 File — File contents include the full text of study instructions, data and analytic strategy supplementary details, and supplementary models. (PDF) [file pone.0222724.s001.pdf]

Supporting Information

for

Group leaders establish cooperative norms that persist in subsequent interactions

Ashley Harrell
Duke University
Department of Sociology
ashley.l.harrell@duke.edu

Contents:

1. Full text of instructions for the public good dilemma
2. Data and analytic strategy supplementary details
3. Supplementary models

Full text of instructions for the public good dilemma

Note: Clarifying headings and notations, not presented in the instructions themselves, are displayed in italics. Instructions shown were presented to the for-pay participant pool (see Data and analytic strategy supplementary details for more information); changes made for the for-credit pool are shown in brackets.

Non-punishment rounds

> In today's study, you will take part in several "investment decisions." Your earnings today will partly depend on how well you understand the instructions. [And as noted below, you may receive your earnings in money today.] So, please read all instructions carefully.
>
> The basic directions are as follows: you will be completing this study in a group of four. Your group members are three other participants currently in the lab. You will not meet these others at any time, nor will you learn any identifying information about them. Likewise, they will not learn any identifying information about you.
>
> At the start of each decision you'll make, you (and each of your group members) get 20 points. You can contribute anywhere from 0 to 20 of these points to a "group fund." Any points you do not contribute to the group fund remain in your personal fund, for you to keep.
>
> **Anything that is contributed to the group fund will be doubled.** Then, the doubled amount will be divided between all four of the members of your group, whether or not they contributed to the group fund. (Similarly, others' contributions to the group fund will be doubled and redistributed equally among all members of the group.) **Your total**

earnings per round are your share of the earnings from the group fund, plus whatever you did not invest. The same goes for other group members.

Points earned over each round will be translated into dollars at the end of the study. [**One randomly selected participant will receive his or her earnings in money at the end of the study.**] **So, the more points you earn, the more money you will receive [, if you are the participant randomly selected to receive your earnings].**

Make sure you have carefully read and understand the instructions. If you have any questions, you may slightly open your door and a research assistant will be with you in a moment. If you understand the instructions, click "Continue".

Let's go over an example.

Imagine that each group member invests all 20 of their points. Thus, there are now 80 points in the group fund (20 points from each of 4 group members).

Since anything in the group fund is doubled, the 80 points become 160 points. Then, the group fund is divided by four (for each of the four group members). So, each group member receives 40 points.

Since everyone invested all of their points to the group fund, each group member finishes the period with 40 points (40 earned from the group fund + 0 kept in the personal fund).

If you have any questions about this example, please slightly open your door and a research assistant will be with you in a moment.

If you understand the example, click "Continue." You will answer several quiz questions to ensure your understanding of the instructions.

Here's another example.

Imagine that each group member invests none of their 20 points. Now, there are zero points in the group fund.

Since there are no points in the group fund to double, and no points to divide by all the group members, everyone earns 20 points from this round: 0 points from the group fund + 20 points kept in the personal fund.

If you have any questions, please slightly open your door and a research assistant will be with you in a moment.

If you understand the example, click "Continue." You will answer several quiz questions to ensure your understanding of the instructions.

Here's another example.

Imagine that three group members invest all 20 of their points, and one group member invests none of their points. Thus, there are 60 points in the group fund (20 points x 3 group members).

The 60 points in the group fund gets doubled to 120 points. Then, the 120 points gets divided into four so that every group member gets 30 points.

The three group members who invested their points end the round with 30 points each (30 points from the group fund + 0 points kept in the personal fund). The group member who invested nothing ends the round with 50 points (30 earned from the group fund + 20 kept in the personal fund).

If you have any questions, please slightly open your door and a research assistant will be with you in a moment.

If you understand the example, click "Continue." You will answer several quiz questions to ensure your understanding of the instructions.

Punishment rounds (presented to participants following nine rounds of the non-punishment phase) [peer punishment condition in brackets]

For the next part of the study, **some of the rules of the task will change.** So, please read all instructions carefully so you understand how the rules will change.

First, **participant IDs have been changed**. You and the others have each been given a new identifying letter. Your previous ID was [previous letter ID]. Your new ID is [new letter ID].

Second, in Part 2 you and the three others will continue to decide how many points, from 0 to 20, you wish to contribute to the group fund, and how many to keep for yourself. **However, now, one group "leader" (chosen at random) [each group member] will have the opportunity to deduct points from their fellow group members' earnings following each round.**

Specifically, after each round (that is, after all members' contributions for that round are known), the leader [each person] will be able to deduct points from non-leaders' [their group members’] earnings, if he/she chooses. It costs 1 point to deduct 3 points from another group member. For example, if the leader [a group member] chooses to spend 2 of his/her points to deduct earnings from Participant M, Participant M's earnings will be reduced by 6 points.

The leader [Group members] can spend anywhere from 0 to 10 of their own points to deduct points from each of their other group members. That is, they may choose not to deduct from a group member's earnings. Or, they may choose to spend some of their own points (up to 10) to deduct points (up to 30) from another group member.

No one can deduct points from the leader, and whoever receives the leader position will have that position for the remainder of the study. In a moment, you and the others will find out who has received the leader position. *[Paragraph not shown in peer punishment condition.]*

Make sure you have carefully read and understand the new rules. Then, click "Continue".

One-shot decision (presented to participants following nine rounds of the punishment phase) [peer punishment condition in brackets]

For the next decision task of today's study, you and your group members will again begin with 20 points and can contribute any portion of these points to the group fund. Any points you do not contribute to the group fund remain in your personal fund, for you to keep, and anything contributed to the group fund will be **doubled** and divided between all four of the members of your group.

Unlike the last task, you will make this decision exactly **once** and you will **not** receive **any** feedback on others' decisions and earnings for this task. That is, you won't see any information about what the others contributed, or how much you earned, unlike you did in the previous rounds.

Relatedly, since you will not see others' decisions or earnings, **the leader [you and the others] will not have the opportunity to make deductions from anyone.** Your earnings from this task will be added to your earnings from the previous task [, which may be paid to you at the end of today’s study, if you are the randomly selected participant to get paid].

If you understand the instructions, click "Continue."

Data and analytic strategy supplementary details

Participants

As briefly noted in the main text, participants were recruited from two separate, mutually exclusive participant pools at a United States university. One pool included students enrolled in introductory courses which offered course credit for participation (those who do not wish to participate in research studies for credit, or who are not yet 18, are offered an alternative to earn this course credit). The second pool was students at least 18 years of age who indicated they were interested in completing research studies in exchange for payment, at about $15 per hour. Students could not belong to both pools.

Each four-person group in the study consisted of either four participants from the for-credit pool (N = 34 groups) or four participants from the for-pay pool (N = 21 groups). The consent process and instructions varied slightly across pools because of the difference in incentives. Specifically, those in the for-pay pool were told that participants in the study session would be paid based on their earnings in the study, ranging from $10 to $15. Those in the for-

credit pool were told that all participants in the study session would receive credit in their courses, and one randomly chosen participant (who would not be selected until the end of the study) would also be paid based on their earnings, ranging from $8 to $10. The lower payment range for the for-credit pool was because the rules of the shared for-credit pool caps participant payments, if any are given, at $10. (This is done to prevent studies that pay from becoming significantly more popular among the students in the pool, given that the pool is a shared resource used by many researchers at the university, and many of the offered studies do not involve payment beyond the course credit.)

The main text reports on contributions in the non-punishment phase and the punishment phase. Adding a term for pool type to those models does not alter results, nor is it predictive of the key measures, despite the small difference in incentives. Models including terms for pool type and other controls are reproduced in the Supplementary Models section below (see Table A for contributions in the non-punishment phase and Table C for contributions in the punishment phase).

Analytic strategy

All models reported in the main text, along with the supplementary models below, use multilevel linear modeling (*lme4*, *lmerTest*, and *boot* packages in R) with random intercepts for the group and the participant (for three-level models) or for the group (two-level models). For all models I use maximum likelihood estimation (ML), allowing for comparability across models (29). Re-running the models using restricted maximum likelihood estimation (REML) does not alter the interpretation of any results (models available from the author). A data file and codebook for replicating the analyses is available via the Open Science Foundation: https://osf.io/9ma4b/.

Supplementary models

Table A. Contributions in the non-punishment phase.

	**Model 1**		**Model 2**		**Model 3**	
	B (SE)	*95% Bootstrap CI*	*B (SE)*	*95% Bootstrap CI*	*B (SE)*	*95% Bootstrap CI*
Leader condition	-1.42 (1.30)	-4.19, 1.04	-1.80 (1.36)	-4.66, .86	-1.75 (1.36)	-4.57, .91
Round	-.36 (.04)***	-.44, -.28	-.40 (.06)***	-.52, -.28	-.40 (.06)***	-.52, -.28
Leader condition x Round			.08 (.08)	-.09, .24	.08 (.08)	-.09, .24
Male					.04 (.58)	-1.07, 1.25
Age					.40 (.22)+	-.06, .82
Credit Pool					.34 (1.37)	-2.39, 3.11

**Intercept**	10.09 (.95)***	8.31, 11.99	10.28 (.97)***	8.45, 12.21	2.24 (4.62)	-6.65, 12.01
**Variance Components**						
Level 1	22.38 (4.73)		22.37 (4.73)		22.37 (4.73)	
Level 2	10.66 (3.26)		10.66 (3.27)		10.39 (3.22)	
Level 3	19.96 (4.47)		19.96 (4.47)		19.97 (4.47)	

Note: + $p < .10$, * $p < .05$, ** $p < .01$, *** $p < .001$. Multilevel linear regression model (maximum likelihood estimation) with random intercepts for participant and group. N = 1980 rounds (nested in 220 participants nested in 55 groups). The reference category for Peer punishment condition is Leader condition; for Male, Female, and for Credit Pool, Payment Pool.

Table B. Contributions in the final round of the non-punishment phase vs. the first round of the punishment phase.

	**Model 1**		**Model 2**		**Model 3**	
	B (SE)	*95% Bootstrap CI*	*B (SE)*	*95% Bootstrap CI*	*B (SE)*	*95% Bootstrap CI*
Leader condition	-.45 (1.42)	-3.30, 2.40	-.95 (1.49)	-3.79, 1.95	-.89 (1.48)	-3.70, 1.97
Round one of punishment phase	2.79 (.44)***	1.93, 3.72	2.29 (.62)***	1.05, 3.53	2.29 (.62)***	1.05, 3.53
Leader condition x Round one of punishment phase			.98 (.87)	-.79, 2.70	.98 (.87)	-.79, 2.70
Male					.71 (.70)	-.67, 2.14
Age					.44 (.27)	-.10, .94
Credit Pool					.47 (1.50)	-2.52, 3.48
**Intercept**	7.21 (1.04)***	5.22, 9.17	7.46 (1.06)***	5.41, 9.49	-1.56 (5.54)	-11.96, 9.72
**Variance Components**						
Level 1	21.01 (4.58)		20.89 (4.57)		20.89 (4.57)	
Level 2	8.93 (2.99)		8.99 (3.00)		8.53 (2.92)	
Level 3	22.86 (4.78)		22.86 (4.78)		22.61 (4.76)	

Note: + *p* < .10, * *p* < .05, ** *p* < .01, *** *p* < .001. Multilevel linear regression model (maximum likelihood estimation) with random intercepts for participant and group. Only behavior in round nine of the non-punishment phase and round one of the punishment phase are included. N = 440 rounds (nested in 220 participants nested in 55 groups). The reference category for Round one of punishment phase is Round nine of non-punishment phase. The reference category for Peer punishment condition is Leader condition; for Male, Female, and for Credit Pool, Payment Pool.

Table C. Contributions in the punishment phase.

	**Model 1**		**Model 2**		**Model 3**	
	B (SE)	*95% Bootstrap CI*	*B (SE)*	*95% Bootstrap CI*	*B (SE)*	*95% Bootstrap CI*
Leader condition	-.08 (1.54)	-3.34, 2.91	1.19 (1.57)	-2.05, 4.20	1.16 (1.56)	-2.04, 4.11
Round	.09 (.03)**	.03, .16	.22 (.05)***	.13, .31	.22 (.05)***	.13, .31
Leader condition x Round			-.25 (.06)***	-.38, -.13	-.25 (.06)***	-.38, -.13
Own contribution in the non-punishment phase	.07 (.02)**	.03, .11	.07 (.02)**	.03, .11	.07 (.02)**	.03, .11
Group average contribution in the non-punishment phase	-.04 (.04)	-.12, .04	-.04 (.04)	-.11, .04	-.04 (.04)	-.11, .04
Male					1.31 (.52)*	.32, 2.39
Age					.10 (.20)	-.31, .48
Credit Pool					-1.38 (1.59)	-4.51, 1.88
**Intercept**	9.90 (1.16)***	7.70, 12.21	9.21 (1.17)***	6.98, 11.53	7.61 (4.27)+	-.67, 16.49
**Variance Components**						
Level 1	13.62 (3.69)		13.50 (3.67)		13.50 (3.67)	
Level 2	9.11 (3.02)		9.13 (3.02)		8.66 (2.94)	
Level 3	30.03 (5.48)		29.84 (5.46)		29.30 (5.41)	

Note: + *p* < .10, * *p* < .05, ** *p* < .01, *** *p* < .001. Multilevel linear regression model (maximum likelihood estimation) with random intercepts for participant and group. N = 1980 rounds (nested in 220 participants nested in 55 groups). The contribution variables range from 0

to 20. The reference category for Peer punishment condition is Leader condition; for Male, Female, and for Credit Pool, Payment Pool.

Table D. Earnings after accounting for punishment sent and received.

	Model 1		Model 2	
	B (SE)	*95% Bootstrap CI*	*B (SE)*	*95% Bootstrap CI*
Leader condition	7.79 (2.28)**	2.95, 12.15	7.88 (1.76)***	4.13, 11.26
Round	.27 (.07)***	.13, .40	.14 (.06)*	.03, .26
Own contribution in the punishment phase			-.37 (.04)***	-.46, -.29
Group average contribution in the punishment phase			1.59 (.08)***	1.44, 1.75
Own contribution in the non-punishment phase	-.04 (.04)	-.11, .04	.03 (.04)	-.03, .10
Group average contribution in the non-punishment phase	.17 (.07)*	.03, .31	.04 (.07)	-.09, .16
Male	-1.46 (.51)**	-2.44, -.43	-.98 (.49)*	-1.92, .03
Age	.43 (.20)*	.01, .83	.42 (.19)*	.02, .79
Credit Pool	-1.65 (2.36)	-6.36, 3.03	-.12 (1.83)	-3.76, 3.51
**Intercept**	11.76 (4.56)*	2.69, 21.08	-.91 (4.22)	-9.20, 7.74
**Variance Components**				
Level 1	52.40 (7.24)		43.55 (6.60)	
Level 2	3.89 (1.97)		4.06 (2.01)	
Level 3	69.05 (8.31)		40.23 (6.34)	

Note: + $p < .10$, * $p < .05$, ** $p < .01$, *** $p < .001$. Multilevel linear regression model (maximum likelihood estimation) with random intercepts for participant and group. N = 1980 rounds (nested in 220 participants nested in 55 groups). Contribution variables range from 0 to 20. The reference category for Peer punishment condition is Leader condition; for Male, Female, and for Credit Pool, Payment Pool.

Table E. Leader behaviors in the previous round predict follower cooperative behavior in the next round *within* the institution.

	Model 1		Model 2	
	B (SE)	*95% Bootstrap CI*	*B (SE)*	*95% Bootstrap CI*
Own contribution	.54 (.04)***	.47, .61	.44 (.05)***	.34, .54
Leader’s contribution	.30 (.03)***	.23, .37	.18 (.05)***	.09, .27
Prosocial punishment received	.69 (1.13)	-1.54, 2.92	.21 (1.15)	-2.02, 2.48

Antisocial punishment received	-2.31 (3.85)	-9.90, 5.44	-3.98 (3.91)	-11.44, 3.86
Group average contribution			.30 (.08)***	.14, .47
Own contribution in the non-punishment phase	.07 (.03)*	.01, .13	.07 (.03)*	.01, .14
Group average contribution in the non-punishment phase	.05 (.05)	-.05, .14	.01 (.05)	-.09, .10
Round	.05 (.06)	-.07, .18	.00 (.06)	-.12, .14
Male	.52 (.33)	-.14, 1.18	.56 (.34)	-.13, 1.23
Age	-.11 (.15)	-.42, .19	-.07 (.15)	-.38, .22
Credit Pool	-.41 (.47)	-1.37, .48	-.13 (.38)	-.85, .59
**Intercept**	2.76 (3.01)	-3.35, 8.76	1.44 (3.08)	-4.52, 7.46
**Variance Components**				
Level 1	12.88 (3.59)		12.84 (3.58)	
Level 2	.00 (.00)		.34 (.59)	
Level 3	.64 (.80)		.00 (.00)	

Note: + *p* < .10, * *p* < .05, ** *p* < .01, *** *p* < .001. Multilevel linear regression model (maximum likelihood estimation) with random intercepts for participant and group. All variables denote behavior in the current round (round *r*); the dependent variable is contribution in the next round (round *r* + 1). This model contains groups in the leader condition only. The peer punishment condition, the leaders in the leader condition, and round nine behavior (because behavior in the next round does not exist in round nine) are omitted from analyses. N = 672 rounds (nested in 84 participants nested in 28 groups). Contribution variables range from 0 to 20; punishment received variables are a proportion out of the total punishment that could have possibly been received and range from 0 to 1. The reference categories are Female and Payment Pool. These models correspond to the Table 1 models in the main text, but include additional control terms for participant characteristics (pool type, age, and gender).

Table F. Leaders' behaviors are more influential in promoting their group members' cooperation in the next round, compared to peers' behaviors in peer punishment institutions.

	**Model 1**		**Model 2**		**Model 3**	
	B (SE)	*95% Bootstrap CI*	*B (SE)*	*95% Bootstrap CI*	*B (SE)*	*95% Bootstrap CI*
Own contribution	.70 (.02)***	.65, .74	.69 (.02)***	.64, .73	.68 (.02)***	.64, .73
Selected group member's contribution	.20 (.02)***	.15, .24	.16 (.03)***	.11, .22	.16 (.03)***	.11, .22
Leader condition	-.26 (.29)	-.82, .32	-1.03 (.47)*	-1.95, -.05	-1.11 (.49)*	-2.07, -.09

Leader condition x Selected group member's contribution			.07 (.03)*	.00, .14	.07 (.04)*	-.00, .14
Prosocial punishment received	2.95 (.87)***	1.26, 4.69	2.73 (.87)**	1.01, 4.49	1.65 (1.49)	-1.20, 4.73
Antisocial punishment received	-.43 (1.63)	-3.63, 2.72	-.44 (1.62)	-3.63, 2.71	-.68 (1.82)	-4.26, 2.88
Prosocial punishment received x Leader condition					1.45 (1.69)	-1.95, 4.75
Antisocial punishment received x Leader condition					.51 (4.16)	-7.72, 8.56
Own contribution in the non-punishment phase	.10 (.02)***	.05, .14	.10 (.02)***	.06, .14	.10 (.02)***	.05, .14
Group average contribution in the non-punishment phase	-.03 (.03)	-.10, .04	-.03 (.03)	-.10, .04	-.03 (.03)	-.09, .04
Round	.04 (.04)	-.05, .13	.05 (.04)	-.04, .14	.05 (.04)	-.04, .14
Male	.37 (.23)	-.08, .84	.39 (.23)	-.07, .85	.40 (.23)+	-.05, .87
Age	.01 (.08)	-.16, .18	.00 (.08)	-.16, .18	-.01 (.09)	-.17, .17
Credit Pool	.10 (.32)	-.52, .75	.15 (.32)	-.48, .81	.13 (.33)	-.51, .80
**Intercept**	.18 (1.76)	-3.36, 3.60	.58 (1.77)	-3.06, 4.10	.86 (1.80)	-2.85, 4.49
**Variance Components**						
Level 1	12.44 (3.53)		12.39 (3.52)		12.36 (3.52)	
Level 2	.00 (.00)		.00 (.00)		.00 (.00)	
Level 3	.56 (.75)		.56 (.75)		.61 (.78)	

Note: + *p* < .10, * *p* < .05, ** *p* < .01, *** *p* < .001. Multilevel linear regression model (maximum likelihood estimation) with random intercepts for participant and group. All variables

denote behavior in the current round (round *r*); the dependent variable is contribution in the next round (round *r* + 1). The "selected group member" is either the leader (in the leader condition) or a randomly chosen peer, held constant across rounds (peer punishment condition). The selected group members themselves (leaders or the randomly chosen peer), as well as round nine behavior (because behavior in the next does not exist in round nine) are omitted from analyses. N = 1320 rounds (nested in 165 participants nested in 55 groups). Contribution variables range from 0 to 20; punishment received variables are a proportion out of the total punishment that could have possibly been received and range from 0 to 1. The reference categories are Female and Payment Pool. These models correspond to the Table 2 models in the main text, but include additional control terms for participant characteristics (pool type, age, and gender).
